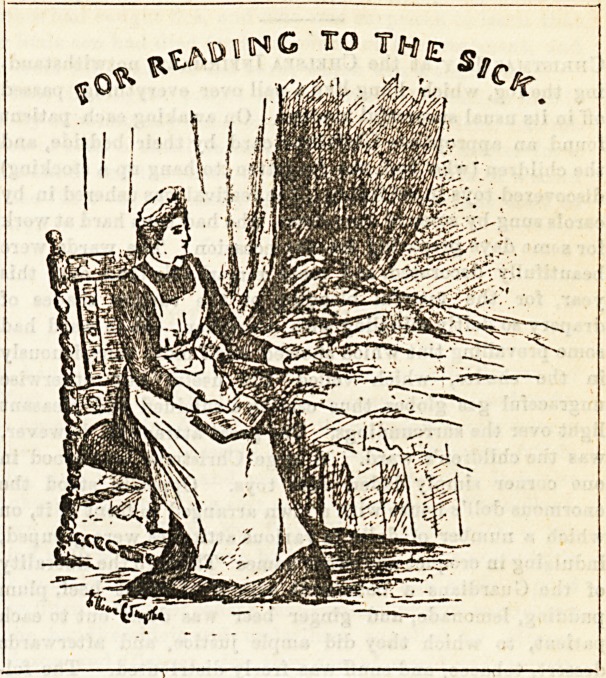# The Hospital Nursing Supplement

**Published:** 1892-01-23

**Authors:** 


"The Hospital" Nursing Mirror.

BEING THE EXTRA NURSING SUPPLEMENT OF "THE HOSPITAL" NEWSPAPER.

Contributions for this Supplement should be addressed to the Editor, THE HOSPITAL, 140, Strand, London, W.C., and should have the word "Nursing" plainly written in left-hand top corner of the envelope.

### En Passant.

THE LATE DUKE OF CLARENCE.—The nurses at the following hospitals, together with private nurses, have contributed to the purchase of the Ionic Cross of violets and lilies of the valley, which was placed as a nurses' tribute of respect and loyalty on the coffin of His Royal Highness the Duke of Clarence and Avondale:—St. Thomas's Hospital, S.E.; Guy's Nursing Institution; Fitzroy House, Fitzroy Square; Paddington Infirmary; Seamen's Hospital; Mildmay Nursing House; Workhouse Infirmary, Hampstead; Home for Sick Children, Sydenham; Leeds Trained Nurses' Institution; General Infirmary, Chester; City Asylum, Birmingham; Mitchell Home, Torquay; County Asylum, Rainhill; Nursing Institute, 4 and 5, Henrietta Street, W.C.; Royal Hants County Hospital, Winchester; The Infirmary, St. George's-in-the-East; Borough Fever Hospital, Leeds; London Association of Nurses, 123, New Bond Street; Kent Nursing Institution, West Malling; London Hospital; Nurses' Home, Fakenham, Norfolk; Bristol Nurses' Training Institution; Nottingham and Notts. Nursing Association; the Workhouse Infirmary, Shoreditch; County Asylum, Northampton; Miss A. W. T. Menck; Mrs. E. A. Tom; Miss E. J. Proviss; and Miss Florence Newton, Wolverhampton Eye Infirmary.

AN EXPRESSION OF SYMPATHY.—The nurses of all countries desire to express their sympathy with the Princess of Wales in her great grief for the loss of her eldest son. The Princess, called back from her holiday to nurse Prince George during typhoid fever, had no time to rest after the strain of her younger son's illness before the Duke of Clarence was attacked with pneumonia following influenza. The attack was brief and fatal, and universal sympathy is felt with the Princess, who, herself of a tender and loving nature, was ever ready to sympathise with others. We have thought it better not to print the letters sent us on this subject; grief so recent and so sore is almost too sacred for condolence, and this short note, we know, expresses the respectful, but deep sympathy which all nurses feel with the Princess of Wales in her great sorrow.

FROME NURSES' HOME.—The eighth annual meeting of Governors of this home was held on January 7th, the Rev. W. A. Duckworth in the chair. The receipts from donations and subscriptions amount to £172 19s. 8d., showing an increase of £38 5s. 8d. upon last year's figures. The Treasurer's account for the year shows a balance in hand of £1 7s. 10d. The table of cases and results shows an increase under the head of "District Nursing" (that is, of attendance on labourers and their families; mechanics and their families; the aged and indigent), of thirteen cases; there had been a decrease in class C (that is of patients who pay a small sum weekly for attendance) of five cases; and an increase in "Private Nursing," of paying patients, of eight cases. There are now eight nurses at work. Miss Briggs herself does a full share of district nursing. Several of the Governors present expressed their personal obligations to the nurses connected with the home.

STARTING DISTRICT NURSES.—In August the District Nursing Association at Stockton was started; and in twenty weeks the nurses have attended 200 cases, the Lady Superintendent taking part in the practical work. There is an energetic Committee, and the reports of the work are submitted monthly to the workmen of the town. This is an important point in a place like Stockton, for the nurses had to win their way into households where nurses were utterly unknown.

THE RELIGIOUS QUESTION.—At the late quarterly meeting of the Norfolk and Norwich Hospital, it was announced that subscriptions to the amount of nearly £100 had been withdrawn because the Lady Superintendent had joined the Roman Catholic Church, and, on doing so, had not resigned. Mr. Duckett, in a very sensible letter on the subject says:—"But it has been said that for the sake of the Hospital the Lady Superintendent ought to resign, and that the institution has suffered, and will suffer, through her retaining her position. Surely there is some mistake about this. Has the ability which she has displayed for fourteen years, has her prudence, her firmness, her ladylike comportment deserted her because she has become a Roman Catholic? Let the medical staff, the nurses, the visitors, the Hospital Sunday Fund say, No; what may injure this great institution, though I still hope it will not, is the mistaken policy which I have ventured to call the unfounded prejudice of those who have or may see fit to withdraw or curtail their subscriptions." It is a strange Christianity which denies to a Catholic any more than to a Protestant the right to minister to the sick. We would point out that in the best hospitals there are no religious restrictions. Also the Nurses' Co-operation has several Catholic nurses on its staff, and has taken some trouble to procure a prominent Catholic as a member of the Committee.

THE NURSES' CO-OPERATION.—On Tuesday the first annual meeting of this Association was held, Dr. Broadbent in the chair. Before the formal business commenced, the Chairman rose and proposed a vote of sympathy with the Prince and Princess of Wales, and with the Queen. Very quietly and gravely the vote was passed, all knowing that Dr. Broadbent himself had been deeply tried by the death of the Duke of Clarence, and all feeling that under the circumstances it was specially kind of him to take the chair. The report stated that during the year 1,015, nurses had applied to join the society, out of which 185 had been accepted. During eleven months over a thousand cases had been attended by the nurses. The financial statement was eminently satisfactory as at the end of the first year the Co-operation is more than paying its way. In rising to move the adoption of the report, Dr. Broadbent said he must first express the pleasure it gave him to preside at the annual meeting of such an excellent association; he then went on, in a charming speech, to praise the methods of the Co-operation, and to give to all nurses due merit as handmaidens to the medical profession. Mr. Burdett seconded. Mr. Henry Kimber, M.P., proposed a vote of thanks to the Committee and officers for their labours during the past year; Mr. Pitts seconded. Dr. Hadden proposed a vote of thanks to Dr. Broadbent for presiding, which Miss Wilson seconded, and then the very short but satisfactory meeting was over. Amongst those present were the Baroness von Hugel, Sister Leigh, General Swanston, Mr. Albert Napper, and other friends of the Co-operation. Letters of apology for non-attendance were read from Lord Crewe, Sir William Moore, the Hon. Roden Noel, Mr G. B. Samuelson, M.P., Mrs. William Black, Mr. Bryant, Dr. Walmsley, Dr. Savile, and others. Great regret was felt at the absence of Miss Hicks, Mr. Cheston, and Dr. Goodhart, owing to the prevailing epidemic.

### Lectures on Surgical Ward Work and Nursing.

By ALEXANDER MILES, M.D. (Edin.), F.R.C.S.E.

### LECTURE XLII.—INSTRUMENTS USED IN LITHOTOMY.

THE operation known as Lithotomy consists in opening into the bladder for the purpose of removing from it a "calculus" or stone. This opening may be made either just above the pubis in the middle line—"supra pubic lithotomy"—or through the perineum—"perineal lithotomy"—and when in this situation the incision may be "median," or much more frequently is "lateral." Obviously the first step in this procedure is to determine whether or not a stone is present. This, of course, is largely decided by the symptoms and history of the patient, but cannot be made certain without the use of an instrument called (*a*) the "Lithotomy Sound." Of these there are several varieties, the more commonly used being (1) Liston's Sound, which consists of a solid steel stem with a curve, somewhat smaller than that of a bougie. The handle is

FIG. 4A.

FIG. 5B.

broad, and flattened out laterally; (2) Lister's Sound is made on the same model as the bougies of his name (*vide* page 74); (3) Sir Henry Thompson's Sound is made of plated silver, with a straight stem and a very short, sharply-curved beak, which permits of the instrument being freely moved about inside the bladder, a proceeding which is further facilitated by the handle being small, rounded, and fluted, so as to admit of fine manipulations being carried out; (4) some other sounds, such as Erichsen's, Teevan's, &c., are hollowed out to permit of fluid being introduced into, or withdrawn from the bladder during the operation of sounding.

The presence of a stone being demonstrated, and an operation considered necessary, after the usual preparations for anæsthesia, &c., the patient has to be placed in what is known as the "lithotomy position," *i.e.*, with the legs flexed on the thighs, and the thighs on the abdomen, the knees widely separated so as fully to expose the perineum. The sole of the foot is placed in the palm of the corresponding hand, and fixed there by means of a soft but strong bandage.

As it is of great importance that the patient should be kept steadily and evenly in this position all the time of the operation, an apparatus known as (*b*) Clover's Crutch, has been devised to facilitate this. It consists of a transverse bar, which may be lengthened or shortened at will by a

FIG. 6.

telescopic arrangement, fitted at each end with a circular padded strap. These straps are adjusted to the legs just below the knee, and the transverse bar keeps the knees apart. A long strap passes from one end of the transverse bar, up across the same shoulder, then crosses the back and comes out over the front of the opposite groin to be fixed to the other end of the bar. In this way the double flexion of the legs is kept up, and the desired position maintained.

The instruments used in the operation itself are (1) Lithotomy knife. Many varieties of this knife have been introduced at different times to render the dangers of the operation less, but as Mr. Ericsen says: "They simply seek to

FIG. 7.

supply by mechanical means that safety in the deeper incisions which may as readily be secured by a broad bladed, straight-backed scalpel, if properly guided by a hand that is ordinarily skilful." The two patterns that are most commonly used are (*a*) Liston's, which has a cutting edge only in the anterior two-thirds, and a sharp point; and (*b*) Fergusson's, which is probe pointed.

Now, in order to open at once into the bladder it is necessary that the surgeon should have some guide, and this is furnished in the shape of (2) the Lithotomy staff. This is a large, curved steel instrument, with a broad, roughened handle. Throughout its lower half or two-thirds it has a deep groove on its left side, and it is this groove which guides the surgeon into the bladder. The instrument is passed in the usual way, and held in position by an assistant. The

FIG. 8.

surgeon then feels it through the skin of the perineum, and cutting boldly down upon it, enters the knife into the groove and runs it straight home into the bladder. Of this instrument, again, there are several varieties (*a*) Chesselden's staff, the oldest and most generally used; (*b*) Buchanan's, which has the lower part set at a right-angle to the stem; and (*c*) Chiene's, in which the right-angle of Buchanan's is rounded off.

The next instrument used in the operation is (3) the Lithotomy forceps. These are large, strong forceps with a scissors joint, the blades being spoon-shaped, roughly serrated on their hollow surfaces, and often lined with linen or wash-leather, to prevent the stone slipping. The handles have a ring on one side for the thumb, and a loop on the other into which the fingers fit. Most forceps are straight, but curved ones are often found useful. Sometimes the stone is more conveniently removed by (4) the Lithotomy scoop than by forceps. This instrument is practically a single blade of the forceps, which may or may not be roughened, set in a handle (Fig. 4A), or it may be double-ended. The finger of the surgeon acts as the second blade of the forceps. The stone having been removed, it is necessary to drain the bladder through the wound in the perineum for some days, and this is done by the use of (5) the Lithotomy tube, which is a short, stiff tube of gum elastic, with a terminal aperture, and provided with silver rings with which to tie it into the bladder. It is of great importance that the tube should be stiff, because it is sometimes necessary to plug the wound tightly to stop bleeding, and if a soft flexible tube be used, it will be occluded, and the drainage of the bladder interfered with. To facilitate this plugging of the wound, the tube is sometimes "petticoated" (Fig. 5B), that is, surrounded by a piece of linen firmly tied round it near its internal end, and hanging loosely round the rest of it. Into the cul-de sac thus formed, wool or lint is tightly packed, and so pressure is exerted on the bleeding vessel. In this operation of Lithotomy, it is necessary to cut widely into the substance of the prostate gland which surrounds the most internal part of the urethra. This may be done with the knife in making the incision into the bladder, and the cut enlarged as far as needful on the way out, or a special instrument known as (6) the "Gorget" may be employed. The small probe-pointed button at the point of the gorget is fitted into the groove in the staff, and as the instrument is pushed home into the bladder, its cutting edge incises the substance of the prostate gland.

For the supra-pubic operation of lithotomy several additional instruments are required. The bladder has to be filled with some tepid antiseptic lotion, *e.g.*, boracic acid. This may be done through an ordinary catheter, by means of a syphon, or with Thompson's hollow sound. Next the bladder is pushed forward as far as possible from the rectum. To effect this an empty india-rubber bag is passed into the rectum, and then filled with lotion, which is retained by a stopcock (Fig. 6). The incision in the abdominal wall is made with an ordinary bistoury, and the parts are opened by the aid of Thompson's Separator (Fig. 7). After the removal of the stone most surgeons drain the bladder through the incision, some using an ordinary rubber or glass drainage tube, furnished with a flat piece of rubber to prevent it slipping right into the bladder. Sir Henry Thompson has a specially constructed india-rubber tube with shield (Fig. 8). Professor Chiene, of Edinburgh, uses a short glass tube, which is made to transfix a piece of large drainage tubing; the latter, lying across the wound, acts as a flange, and prevents the glass tube disappearing into the bladder. This has the advantage of being simple, efficient, and always obtainable.

The illustrations are used by kind permission of Messrs. Arnold and Sons.

INFLUENZA.—A ward has been put apart at St. Thomas's for nurses with influenza, and directly a nurse shows signs of illness she is invalided and carefully nursed. Thus, though there have been many cases, in none have complications ensued. We wish all Matrons and home Sisters took such care of their staff. The above is an example worth following.

### BURDENS.

WE often forget amid the pleasures and comforts of life that we ought not to live to ourselves; but only let health be taken from us and we soon find out how weak and dependent we are upon our fellow creatures; that, in fact, every life hangs upon other lives, and whether we give or take we cannot stand alone.

Some folks boast that they want no help for themselves, and at the same time give their neighbour pretty plainly to understand they may expect none from them. Alas! what a sad and loveless life they lead, only to find out, perhaps when too late, that they have slighted and thrown away what might have been the pleasure and comfort of their lives.

Now that we are sick and powerless to help ourselves, let us think what it would be if we had no one to love and pity us, or wait upon our wants. Among the Greeks and Romans in old times little regard was paid to the old and afflicted, they were looked upon as cumberers of the ground, and their happier brethren passed them by on the other side, just as our Lord tells us the unfeeling did among the Jews, who had no excuse, having been taught better by God Himself; while in heathen lands in our own time, when people can no longer work, they crawl away to hide themselves like a sick animal to die.

How different it is in our Christian land, where men and women of all classes, from the highest to the lowest, vie in alleviating and curing the numerous diseases our flesh is heir to.

Holy Scripture tells us every man must bear his own burden, at the same time we read, "Bear ye one another's burdens," and so fulfil the law of Christ. Yes, the law of Him who bore our griefs and carried our sorrows Himself, who bids us take His yoke upon us, because it is an easy one, and His burden because it is light.

What are the burdens we must carry ourselves? It may be the throbbing head, the wearied limbs, or perhaps a ceaseless round of toil which has borne us nearly to death's door. But has not the loving mother, or sister, or kind nurse smoothed our pillows, calmed our spirits by a gentle touch, and warmed our hearts with the sympathy which came straight from her own?

Is not this, indeed, bearing our burdens for us, and should not we do our best to lighten theirs? The grateful look even when the tongue feels too parched to thank, the striving against restlessness in other stages of illness, and the patient waiting for the attention we need but which has been unavoidably delayed, all these things may seem but trifles, yet they are an immense help to the anxious watcher, and draw us personally nearer to the Christ, who sees all our weak efforts to be like Him. With love He bends over us and says, "Peace be still"; to the weary He whispers, "Come unto Me, and I will give you rest," and to His angels He gives a charge to bear us in their arms into that land of peace and rest where all burdens and pains and griefs shall be done away.

### Keeping Christmas.

CHRISTMAS DAY at the CHELSEA INFIRMARY, notwithstanding the fog, which hung like a pall over everything, passed off in its usual successful manner. On awaking each patient found an appropriate Christmas card by their bedside, and the children (who had not forgotten to hang up a stocking) discovered toys in addition. The festival was ushered in by carols sung by some of the nurses, who had been hard at work for some days preparing for the occasion. The wards were beautifully decorated, and more remarkable than ever this year, for the tasteful blending of the various shades of drapery so deftly mingling with evergreen. Each ward had some prevailing tint which showed itself most conspicuously in the shades, which veiled the useful but otherwise ungraceful gas globes, thus casting a subdued and pleasant light over the surroundings. The great attraction, however, was the children's ward. A large Christmas tree stood in one corner simply laden with toys. Opposite stood the enormous doll's house with a lawn arranged in front of it, on which a number of dolls in various attitudes were grouped, indulging in croquet and other games Through the liberality of the Guardians a bounteous supply of roast beef, plum pudding, lemonade, and ginger beer was dealt out to each patient, to which they did ample justice, and afterwards dessert, tobacco, and snuff was freely distributed. The following day the nurses and other officers of the institution had an evening party, and were entertained afterwards to a sumptuous and much-appreciated repast.

The SAMARITAN FREE HOSPITAL had its entertainment on New Year's Eve. At four o'clock in the afternoon over a hundred persons sat down to tea, and the manner in which the good things provided disappeared spoke well for the appetites of the patients as well as nurses. There were no formalities about the proceedings, and general kindness and courtesy prevailed. After manifesting their appreciation of the generosity shown to them in the most practical manner possible, the company adjourned to Dr. Boulton's ward, which had been fitted up as a concert-room and tastefully decorated with evergreens and flowers. Here a most charming entertainment was given, in which Mr. George Grossmith, Mr. John Thomas, Mr. Ernest Bantock, and other professionals kindly took part. Miss Butler, the Matron, Dr. Granville Bantock, Mr. Stormont Murray, and Mr G. Scudamore, the Secretary, then distributed the gifts given by friends of the institution to each one present, consisting of articles for use and for ornament. The National Anthem brought the proceedings to a close.

On Tuesday evening, 29th ult., the Matron (Miss Cheatle) gave an entertainment to the patients who have been treated in the PAIGNTON COTTAGE HOSPITAL since it was opened (February). Besides patients, there was a large attendance of visitors who subscribe to the hospital. In the ward cleared for the occasion, two tables, one for visitors and the other for patients, were handsomely laid, and here at 5.30, all sat down to a sumptuous repast. Miss Cheatle and Miss Spens presided over the visitors' table, and Miss Baker and Mrs. Anderson looked after the patients. After tea a capital programme was gone through, including songs, recitations, reading, and a step dance, which was greatly enjoyed by all. Amongst those who assisted so ably with songs were the Misses Spens and Miss Lambshead. At the close Dr. Goodridge proposed a vote of thanks to Miss Cheatle for her kindness to the patients, referred to her able management of the hospital, and hoped she might long be Matron thereof. Mr. Waycott seconded, and also spoke in the highest terms of Miss Cheatle. The proceedings were brought to a close by the singing of "God Save the Queen."

AT BEVERLEY COTTAGE HOSPITAL the Matron entertained 50 past and present patients. There was a sumptuous tea, a magic lantern, songs, and finally a Christmas tree and presents for all. A most enjoyable evening was spent.

At WIGAN INFIRMARY Christmas Day began early with carols, the nurses being the choristers. Miss Macentyre provided turkeys and other seasonable fare for dinner, and some of the nurses played and sang during the meal. Altogether, the day was made as happy as possible for the poor sick. On January 7th a most successful concert was held, Lord Balcarres presiding. The sisters and nurses gave all the songs and recitations, and were heartily applauded.

At the annual entertainment to the patients at the SALFORD ROYAL HOSPITAL a large number of the friends of the institution assembled, including Mr. Oliver Heywood, the Rev. Canon Heywood, Mr. N. Shelmerdine, Mr. G. Southam, and Mr. B. Armitage. Christmas trees were provided in both the adult and the children's wards, and the distribution of gifts from these occasioned considerable pleasure. A short musical entertainment was also given. Besides the gifts to the patients, a number of suitable presents were handed to the nurses and the servants by several of the lady visitors.

### District Nurses for Brighton and Hove.

BY THE HON. RODEN NOEL.

THE admirable work done by Miss Josephine Wake in providing nurses for attending the sick poor of Brighton and Hove at their own homes, is well worthy of public support, and stands in much need of pecuniary assistance at the present time. An appeal for this good object was made in THE HOSPITAL for July, 1889; since then the health of Miss Wake has suffered from overstrain, and she is now taking a little needful rest. Nothing is more likely to contribute to her speedy restoration, than a hearty response to her appeal for funds wherewith to carry on her humane work, more especially at the present time when assistance of this kind is so much needed, serious illness being so prevalent. There are now seven nurses working in the different parishes of the two towns. But in order that even this number, a number of course quite inadequate for the need of so large a place, should be maintained, more subscriptions and donations are urgently required, the institution (office, 24, Dorset Gardens, St. James' Street) being dependent on voluntary contributions. Its bankers are the Union Bank, North Street, to which contributions may be sent. In the past year H.R.H. Princess Christian of Schleswig-Holstein has become a Patroness. The first trained nurse was employed in the poorest district of the parish of St. John the Evangelist in the year 1877. In 1889 Dr. W. J. Stephens reported in THE HOSPITAL that there were 60,000 poor inhabitants in Brighton and Hove, not reckoning the outlying villages where similar help is much needed; and I need only add, without being in a position to supply precise statistics that both towns have steadily increased in extent and population since then. Dr. Stephens testifies personally to the good work done by the nurses whom he pronounces thoroughly qualified for their duties by professional training. They undertake about 600 cases per annum, some cases requiring three or four visits daily. "They carry out all necessary sanitary arrangements, doing their utmost to comfort and relieve the suffering; instructing the friends and relatives of the patient in all needful ways, and refusing no office of kindness." The work is entirely unsectarian. I was most favourably impressed by the nurse to whom Miss Wake introduced me. She hopes that a public meeting in aid of the funds of this institution may be held here at no distant date, under the presidency of Dr. Ewart, the Mayor.

### On the Nursing of Children.

### No. II.—EMERGENCIES.

EMERGENCIES often arise in the care and nursing of children, for, as we all know, the most unexpected things happen to, and are perpetrated by, these little creatures.

We may be taking charge of one who is suffering from only a slight ailment, when we are suddenly made anxious by a rise of temperature, or a rash, or a "croupy" cough. In any such fresh development the safest plan, while awaiting the doctor, is to look upon the new symptom as a serious one. Happily it may prove of less consequence than appears at first sight; but in any case, it is wiser not to make light of anything which affects a child's health.

By all means avoid making other people needlessly anxious, and do not let the patient be worried, but give him your most watchful care, and on no account let other children be in the room until it is quite certain that no risk attends their presence there.

Apply promptly such simple remedies as may be necessary, and keep your patient quiet, but avoid the temptation to prescribe medicine. It is often a real temptation, for the child's friends besiege the nurse with the enquiry, "What shall we give him?" and she is well aware that if she named a drug it would be administered without hesitation.

Yes, any "dose" is taken without scruple; but many a mother demurs to nurse's suggestion of the importance of ventilation, and the absolute necessity for maintaining an even temperature in the sick child's room. Even more difficult is the question of food, its quality, the quantity, and the punctuality with which it must be given, &c. And we have to explain, over and over again, that what is harmless in health is often most harmful in sickness.

It is far easier for a nurse to cope with a severe case of illness, when there are hard and fast rules to guide her, than it is for her to meet a sudden call on her own resources.

A thoroughly-experienced district nurse gets used to many and strange things to which a ward nurse is never exposed; and a most useful woman is the aforesaid district nurse, when she has been thoroughly trained in all branches of her profession; but we fear there are still some nurses whose imperfect training renders their real ignorance more dangerous than that of any other class of women. It is terrible to think of precious lives left in these ignorant hands.

When an accident or a fresh attack of illness occurs to a child already in our care, we have the immense advantage of an intimate knowledge of its disposition and general health, and naturally this helps us in the emergency to guess with some correctness how much it is desirable to do whilst awaiting the doctor.

When, however, a private nurse or a district nurse is suddenly called to the aid of a stranger child, she has more difficulties to contend with. She needs as much tact as intelligence when she probably finds the poor little victim already surrounded by a group of kindly, but injudicious sympathisers, and she has to reduce the number of these immediately and without letting anyone feel slighted. Then she must proceed, without delay, to do what is best for the patient.

In cases of burns or scalds, which are amongst the most serious of the mishaps which befall children, there is the terrible fright of the little one, as well as the alarm of his friends, to add to the trouble. This is a perfectly natural condition of things, but it must not be given way to, as the visible distress of his elders must add to the child's terror, just as the injudicious and hasty stripping off of his clothes increases his pain and his danger.

All exposure should be strictly avoided, although, probably, few people would copy the wretched mother, who pumped upon her tiny child one bitter January day when his clothes had caught fire, and who was surprised to learn that her little son had died from the effects of her treatment, and not from the results of the burn, which was a slight one.

Many intelligent people fail to grasp the danger that is run by exposing any child to cold after an accident, but the shock of even a small casualty is not a slight matter for a little child, and the shock to the system of a burn or a scald is the first of the many dangers attending that class of injury.

*(To be continued.)*

### Hints for Nurses.

WE have received from Messrs. Robinson and Cleaver, of Belfast, specimens of their nurses' aprons, collars, and cuffs. The apron is made of good white linen, with a bib, a large pocket, and straps crossing on the back. It is very full in shape, neat in make, and prettily hem-stitched. It is also the cheapest apron of the sort we have ever seen. The collars, known as the "Sister Eva," are Eton shape, with a tab behind with which they can be held down; there are outside cuffs to match. These also are very cheap, and made of good white linen. It is, of course, unnecessary to give particulars of the handkerchiefs and underlinen supplied by Messrs. Robinson and Cleaver, for they are of world-wide reputation.

Nurses frequently have to see that a convalescent patient procures woollen underclothing to avoid chance of a relapse. In private nursing patients are often found whose objection to woollen garments is their colour and their ugliness. This can be overcome by getting the pretty white woollen underclothing provided by Edmonds, Orr, and Co., of 47, Wigmore Street. We saw a trousseau there the other day, most dainty in appearance, most hygienic in principle. All the garments were of wool, merino, or silk, or charming mixtures of silk and wool; they were trimmed with lace, and monograms worked on them in silk. They certainly left nothing to be desired in the way of appearance. But what struck us as the most original article sold by Messrs. Orr was the corset-bodice—such a sensible substitute for the usual stiff corset for young girls or slender women. The corset-bodice is made of elastic merino, or silk, or stockinette. It is supplied with slender flexible bones, and does not lace. It just gives the support that is requisite for comfort without endangering the health by constricting the figure.

### Everybody's Opinion.

*[Correspondence on all subjects is invited, but we cannot in any way be responsible for the opinions expressed by our correspondents. No communications can be entertained if the name and address of the correspondent is not given, or unless one side of the paper only be written on.]*

### AMATEURS ON NURSING.

DORA SCRIMGEOUR writes: I was glad to see the announcement in last week's HOSPITAL of the addition of twelve nurses to the staff of the London Hospital, which you say will enable the Committee to give an extra week's holiday in the year to staff nurses. But why stop short at staff nurses? In 8,992 of the Blue Book for 1890, on Metropolitan Hospitals, I find that Miss Lückes puts the number of nurses absent on their annual fortnight's holiday at ten. In 8,063 of the same book she gives seven or eight as the average number. Surely, then, it would not require more than four extra nurses to allow all having another week, that is, half a fortnight. If I am mistaken, I shall be glad if you will enlighten me on this point. But your remark about my ignorance of hospital affairs is no reply to my argument, which is based on facts given by the hospital authorities. When a proposal of this sort is made by an independent Governor, is it unreasonable to expect that it should receive at least a courteous explanation of its impracticability from the Committee if based on false premises? Of course, everybody interested in the London Hospital will be rejoiced to hear of the welcome addition of twelve nurses to the staff. But do not let it be supposed that these are required simply to give thirty-six women another week's holiday in the year. The Committee might gain the power to do this easily by retaining in the hospital those nurses whom they are in the habit of sending out on private nursing. Why were we not told of this proposed improvement at the Governors' Court? It seems strange that no mention whatever should have been made of it by the Committee, but I can hardly believe that they were unaware of the acquisition of the twelve bed-rooms, although they continue to display an astonishing ignorance as to any details connected with the arrangements for nurses.

### Off Duty.

### Those Three.

"Nothing to pay, mum!" and, with a dump, a square something was set down upon the doorstep, just shaving my toes. Then, the railway-man hurried back to his van which, in a trice, was lumbering away in the distance, a black shadow in the London fog.

I am a single woman of discreet years, an alone-standing lady, as the Germans prettily put it, one of those individuals who, in my own youth, were pointed at, by the ribald, as old maids, a *genus* one never hears mentioned in these days of the feminine millennium. I rule a modest kingdom, my household; I hold a dignified social position, in that state of life in which it has pleased God to place me; in short, I fill a niche in this world, and, although there is no patter of little restless feet about my home, no eager, ceaseless prattle of questioning voices, the sweetest worry a true woman has to endure, still, I lead a tolerably happy life, owing, perhaps, to the various pets that have tyrannised over me, since my boy Ted, the nephew whom I brought up and mothered, left me for a good appointment in Brazil. It is, thanks to him, that such a succession of creatures occupy my heart and home.

When I receive a letter from the boy intimating that a live something will arrive about such a date, I usually mount guard over the front door, attending myself to the knocks and rings, in order to prevent a recurrence of the hysterical scenes with which the maids received Sancho, our monkey, when he arrived. Thus it came about that my toes had such a narrow escape. Gingerly I moved the square package, and was not surprised to find its front was open, with wires across, exposing to view three hapless visitors that had come across the seas to me.

Parrots!

The maids were summoned, and loud was the pity over the appearance of my guests. It was a tight fit for the birds; their unfortunate heads scraped the top of the box, while their tails more than reached the bottom of it. To release the arrivals was short work, and they were soon quite at home in the little conservatory upstairs, where their colouring of scarlet and green matched the trees rather than plants, of geraniums, which were my pride, and among which it was not easy to distinguish the newcomers. Rather to my relief I discovered the parrots were untrained, therefore my maidenly ears were in no danger of being pained by indiscreet language.

It was some little time after the three parrots' arrival that I had one of my periodical luncheon parties, the only form of entertainment in which I indulged. It so happened that I had but two acceptances to my invitations; the retired army-surgeon from the terrace opposite, and a maiden-lady, both of them, perhaps, the most ultra-fastidious acquaintances on my visiting-list, the doctor being shy into the bargain. I felt as we sat down to table a thrill of thankfulness that—my cook being immaculate—there would be no hitch, at least, in the business of the luncheon, to bring down contempt upon me, in the presence of such over-nice, difficult-to-please guests. Everything was proceeding on smooth wheels; we were enjoying ourselves decorously; the doctor was entertaining us with some of his choicest reminiscences of a past military life, when dreadful sounds from above fell, with an awful distinctness, on our ears.

"Close the door, Matilda!" I gasped in a whisper, and my elderly parlour-maid, her face a picture of petrified horror, hastily obeyed.

It was useless. The sounds were redoubled; my cheeks were stained with painful blushes, my eyes glued to the table-cloth. As for my guests, one swift glance had showed me *their* disconcerted embarrassment. There was a silence which I tried to cover with a fit of coughing, echoed by the doctor. In vain! Upstairs, it was plain that some unhappy beings were *in extremis*. I felt myself—such is imagination—on board the Dover packet, where some unfortunates were "doing Channel," as Ted used to say. Picture! the situation; two highly-refined maiden ladies, and a shy, old bachelor listening to those sounds of woe which became louder and more pronounced each moment! At last the climax of our agony was reached; we each felt we could stand the strain no longer.

"Bless my soul, Madam!" All the doctor rose up in the man as the army surgeon sprang to his feet. "I must do something for the unhappy wretches!" and away he rushed, and we ladies at his heels, expecting to find the maids violently ill. The hideous sounds seemed to come from the conservatory of all places, but as we burst in there was a sudden hush. Then deafening peals of jeering laughter broke forth, and my guests, with scared faces, stared round the empty greenhouse. A faint rustle among the overgrown geraniums solved the mystery for me. Those wretched birds, the three parrots, had taught themselves to mimic, with horribly faithful accuracy, the painful sounds to which a rough voyage had accustomed them!

I draw a veil over the effect of the explanation on my fastidious guests who are no longer on my visiting-list, having removed themselves from it. As for those parrots, the three are boarded out with an obliging charwoman, and I tremble each time the South American mail is due over what uncanny visitors it may bring to disturb the decorous equanimity of my home.

### Presentations.

On Christmas Day Miss de Pledge, Matron of the Chelsea Infirmary, was presented by the nursing staff with a beautiful silver cruet stand and butter dish, in token of their good will and regard. A suitable inscription accompanied the gift.

On January 19th, Miss M. M. Belcher, was presented with a writing-table by some of the nurses of the Co-operation. The table bore a brass plate, stating that it was given in acknowledgment of Miss Belcher's great services.

### Appointment.

Totnes College Hospital.—Mrs. Tucker has been appointed Matron of this hospital. She trained at the Clifton Institution in 1884, and has worked for three years for the Exeter Institution. Mrs. Tucker holds good testimonials.

### Notes and Queries.

### Queries.

*H. M.'s Nursing Sisters.*—Will some Sister in the army or navy hospitals kindly tell the Superintendent of a Government hospital abroad what uniform is allowed them, or what the equivalent in money would come to?

### Answers.

*The Nurses' Bed.*—One guinea subscription received from Nurse Parker, and 2s. from Nurse Clarke.

*Oldham Infirmary.*—Through a printer's error, the name of the Matron of this institution was given last week as "Simpson," instead of "Thompson."

*Lady Warden.*—Your letter is written on both sides of the paper.

*E. M.*—We cannot print your verses, we think it wiser to print no communications on the sad occurrence.

## Figures and Tables

**Fig. 4a. f1:**
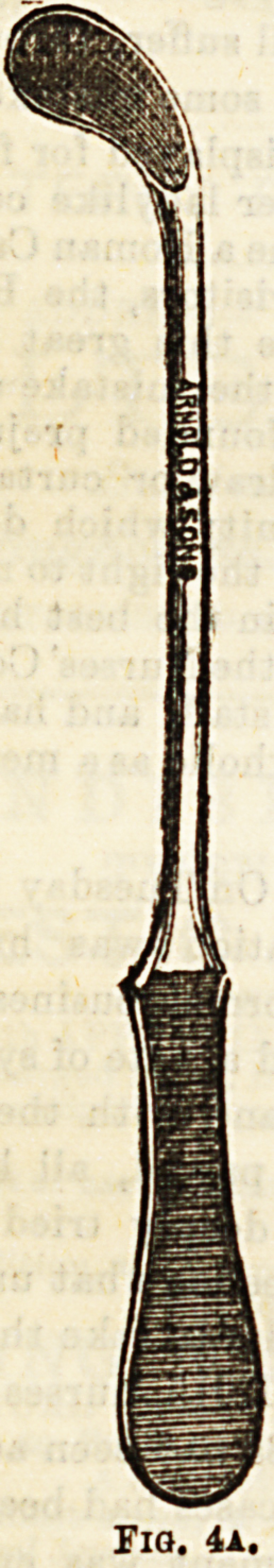


**Fig. 5b. f2:**
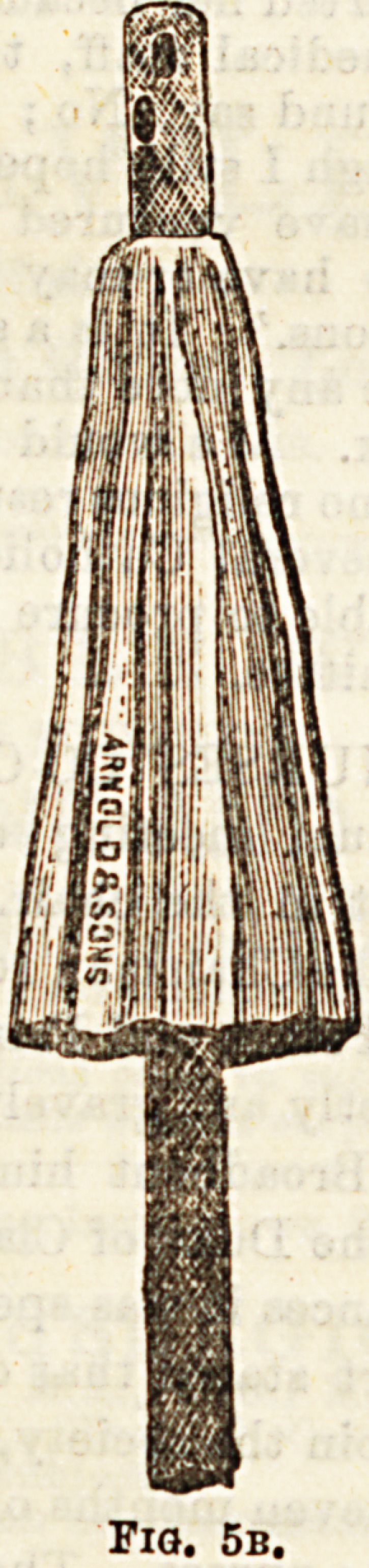


**Fig. 6. f3:**
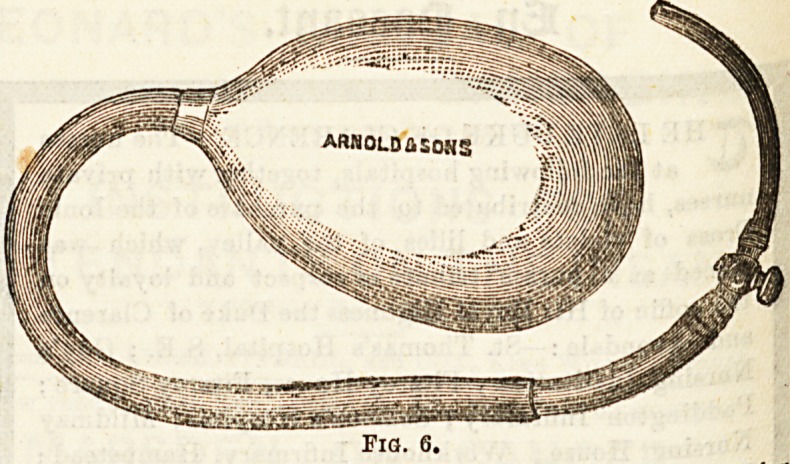


**Fig. 7. f4:**
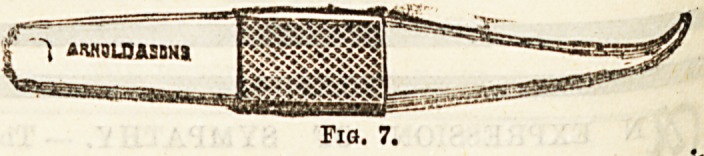


**Fig. 8. f5:**
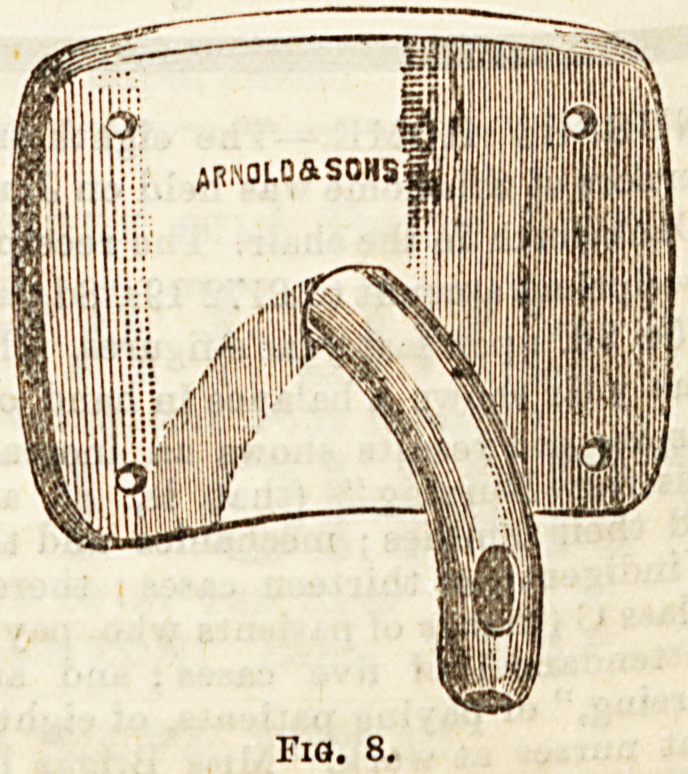


**Figure f6:**